# The Enhanced Liver Fibrosis test is associated with liver-related outcomes in postmenopausal women with risk factors for liver disease

**DOI:** 10.1186/s12876-020-01251-w

**Published:** 2020-04-15

**Authors:** Paul M. Trembling, Sophia Apostolidou, Aleksandra Gentry-Maharaj, Julie Parkes, Andy Ryan, Sudeep Tanwar, Matthew Burnell, Scott Harris, Usha Menon, William M. Rosenberg

**Affiliations:** 1 Division of Medicine, University College London, Institute for Liver and Digestive Health, Royal Free Hospital, Rowland Hill Street, London, NW3 2PF UK; 2 MRC Clinical Trials Unit at UCL, Institute of Clinical Trials and Methodology, Faculty of Population Health Sciences, University College London, 2nd Floor, 90 High Holborn, London, WC1V 6LJ UK; 3 Primary Care and Population Sciences Academic Unit, Faculty of Medicine, University of Southampton, Level C, South Academic Block, University Hospital Southampton, Southampton, SO16 6YD UK

**Keywords:** Alcohol-related liver disease, Non-alcoholic fatty liver disease, Obesity, Liver fibrosis

## Abstract

**Background:**

Chronic liver disease (CLD) is usually asymptomatic but earlier detection is critical to permit life-saving interventions for those at risk due to high alcohol consumption and increased body mass index (BMI). The aim of this study was to estimate the association between the Enhanced Liver Fibrosis (ELF) test and liver-related events (LRE) and its performance in predicting LRE in postmenopausal women with risk factors in a nested case-control study within the United Kingdom Trial of Ovarian Cancer Screening (UKCTOCS).

**Methods:**

In a cohort of 95,126 we performed a case-control study measuring ELF in blinded samples from 173 participants with self-reported high alcohol use and / or BMI ≥25 kg/m^2^ comprising all 58 cases who developed LRE and 115 controls matched for age, alcohol and BMI who did not develop LRE during median follow-up of 8.5 years.

**Results:**

Using Cox regression at an ELF threshold of 10.51 hazard ratios (HR) for LRE were 4.88 (95% confidence interval (CI) 2.37–10.03) (unadjusted model) and 4.62 (95% CI 2.12–10.08) (adjusted for deprivation and self-reported hypertension, heart disease, hypercholesterolaemia and diabetes). At a threshold of 9.8 HR for LRE were 2.21 (95% CI 1.22–3.97) (unadjusted model) and 2.18 (95% CI 1.19–4.01) (adjusted). ELF was evaluated as a time dependent variable by generating time-dependent Cox models; HRs at an ELF threshold of 10.51 were 1.94 (95% CI 1.10–3.39) (unadjusted) and 2.05 (95% CI 1.16–3.64) (adjusted) and at a threshold of 9.8 HRs were 1.85 (95% CI 1.09–3.15) (unadjusted) and 1.80 (95% CI 1.04–3.13) (adjusted). Area under the receiver operating characteristic curve for recruitment ELF predicting LRE was 0.58 (95% CI 0.49–0.68), and for second subsequent ELF 0.61 (95% CI 0.52–0.71).

**Conclusion:**

This study demonstrates the association between ELF and CLD in postmenopausal women with risk factors for liver disease, creating the opportunity to intervene to reduce liver-related mortality and morbidity. Although larger studies are required, these results demonstrate the potential of ELF as a prognostic tool in health checks in primary care.

**Trial registration:**

This study is nested in UKCTOCS. UKCTOCS is registered as an International Standard Randomised Controlled Trial, number ISRCTN22488978. Registered 06/04/2000.

## Background

Due to the rising prevalence of chronic liver disease (CLD), in particular driven by non-alcoholic fatty liver disease (NAFLD) and obesity, there is much potential in targeted case-finding strategies in the community in an attempt to identify liver disease. General practitioners should be aware of this group of patients, which is challenging due to lack of symptoms and signs, but need to be given tools to identify liver fibrosis and diagnostic tests for earlier identification of liver disease.

Although hazardous alcohol use is declining in the younger age groups, this is not the case in the over fifties [1]. Particularly worrying is the increasing proportion of women drinking in later life, driven by life events including bereavement, changes in personal circumstances and retirement [2]. Therefore, not only is it important for screening and treatment of alcohol misuse in the community, it is crucial to focus on the older population.

The Enhanced Liver Fibrosis (ELF) test is a non-invasive measure of liver fibrosis that requires a simple blood sample. The ELF test score, indicating the degree of fibrosis, is calculated from measurement of three markers of fibrosis; hyaluronic acid (HA), tissue inhibitor of matrix metalloproteinase-1 (TIMP-1) and aminoterminal propeptide of procollagen type III (PIIINP). The algorithm was initially derived from a cohort of individuals with CLD with a range of aetiologies [3].

In subsequent validation studies, the ELF test has been shown to identify liver fibrosis in patients with NAFLD, primary biliary cholangitis, primary sclerosing cholangitis, chronic hepatitis B and chronic hepatitis C [4–8] and to predict liver-related clinical outcomes [9].

We have previously described the association between increasing body mass index (BMI) and liver-related events (LRE) in a large cohort of women participating in the United Kingdom Trial of Ovarian Cancer Screening (UKCTOCS) [10]. In the present study we aimed to investigate the association of the ELF test with LRE in postmenopausal women with risk factors comprising high BMI and / or high alcohol consumption in a case-control study using the PRoBE design (prospective-specimen-collection, retrospective-blinded-evaluation) [11].

## Methods

### Study population

This population was drawn from primary care records. In the UK the population are encouraged to register with a primary care physician or general practitioner who then holds their “primary (healthcare) care record.” UKCTOCS is a multi-centre UK-based randomised controlled trial investigating the impact of ovarian cancer screening on mortality. Participants were identified at random from primary care and invited to register for the study. Between April 2001 and October 2005, 202,638 postmenopausal women aged 50–74 were recruited in England, Wales and Northern Ireland. Further information on the UKCTOCS study can be found elsewhere [12–15]. The study was nested in UKCTOCS.

### Exposures

We calculated the BMI of each participant using self-reported height and weight (BMI (kg/m^2^) = weight (kg)/(height^2^)(m)), and then categorised BMI according to the World Health Organization’s definitions; normal (< 25 kg/m^2^), overweight (25- < 30 kg/m^2^) or obese (≥30 kg/m^2^). Taking a pragmatic approach, we excluded participants from this study if self-reported height was less than 140 cm or greater than 210 cm, and / or if self-reported weight lay outside the range 25–200 kg. In addition, we excluded participants in whom calculated BMI lay outside the range 16–65 kg/m^2^.

At approximately 3 years after entry to the study participants were asked to report their average current alcohol consumption over a typical week. We converted each response to UK alcohol units on the assumption that that one drink (one measure of spirit, half a pint of cider / beer or one glass of wine) comprised one unit (10 ml or 8 g of pure alcohol) [16]. Responses were categorised as follows; none, less than 1, 1–3, 4–6, 7–10, 11–15, 16–20 or ≥ 21 units. The UK Chief Medical Officer’s (CMO) guidance is to limit weekly alcohol intake to no more than 14 units per week (for men and women) [16]. This threshold falls within the 11–15 units / week category in the UKCTOCS categories, therefore in this study, this category was included in the definition of ‘high alcohol’. Although this may over-estimate ‘high alcohol’ use it ensures women consuming alcohol over the recommended limit are included. We did not include participants in this study who failed to provide information on alcohol use.

### Covariates

Participants in UKCTOCS completed a questionnaire which collected data on heart disease, hypercholesterolaemia, diabetes, hypertension and smoking status. Socioeconomic status was estimated using the Index of Multiple Deprivation 2007 (IMD) [17, 18]. IMD is a measure of deprivation based on the participants’ postal codes. The score is the UK government’s official measure of multiple deprivation and combines a number of indices including income, employment, health, education and crime.

### Follow up

Cancer registrations and deaths in the UKCTOCS population were notified via a ‘flagging’ study with NHS Digital. Hospital inpatient and outpatient episode data were available through linkage of participants to the Hospital Episodes Statistics (HES) database. Each HES record reports a main diagnosis and up to 19 (inpatient admissions) and 11 (outpatient appointments) further diagnoses. Each death record reports the primary cause of death and additional contributory causes recorded on the death certificate. Diagnoses and causes of death were coded in accordance with the International Classification of Diseases, version 10 (ICD-10). Participants in this nested study were followed up until 1 February 2013. Only participants in England were included, due to the availability of their relevant HES data. In an attempt to exclude participants with known liver disease, participants were excluded from the study if a code within our definition of liver disease was assigned to participants prior to the date of the follow-up questionnaire.

### Selection of cases and controls

We performed a case-control study nested in UKCTOCS. Cases comprised all participants in the cohort with risk factors, defined as BMI ≥25 kg/m^2^ and / or self-reported alcohol consumption of ≥11 drinks per week, with a first presentation of an LRE, defined as first presentation of one or more of the following: a hospital admission, outpatient appointment, cancer registration with, or death from, an ICD-10 code of interest. We searched for the following codes: K70 (alcoholic liver disease), K73 (chronic hepatitis) and K74 (fibrosis and cirrhosis). The use of these codes is consistent with other UK studies of cirrhosis [19, 20]. We also searched for K76.0 (other diseases of the liver, including fat). Codes relating to sequelae of decompensated liver disease were also included; I85 (oesophageal varices), Z944 (liver transplant) and C22.0 (hepatocellular carcinoma). In addition to ICD-10 code, death certificates were also searched for any mention of alcoholic liver disease or fatty liver.

Each case was matched to two controls in order to reduce selection bias. Controls were participants with risk factors who did not experience an LRE. The control matching criteria were: age (+/− 5 years) at recruitment, BMI (+/− 2 kg/m^2^), alcohol group and regional trial (recruitment) centre. Cases were not matched for time to spin (the time between sample collection and centrifugation of sample) or storage time of sample.

### Sample collection and serum marker testing

Stored serum samples were retrieved from the UKCTOCS cryorepository for testing. From cases, blood samples selected for his study were those taken at trial recruitment (sample 1) and at two further time points before LRE (samples 2 and 3). Samples taken up to 6 months prior to the event were not included to reduce risk of the liver event itself influencing the ELF score. In controls, blood samples selected were those taken at recruitment and two similar time points to the respective cases.

Samples were collected into Greiner Bio-One gel tubes (Greiner Bio-One Ltd., Stonehouse) at the UKCTOCS trial centres and shipped overnight at room temperature to the central laboratory. Samples were centrifuged at 1500 g for 10 min and the separated serum aliquoted into 10 × 500 μl straws using a semi-automated MAPI platform (MAPI CryoBioSystem, Cryo Bio System, Paris, France). The straws were heat-sealed, barcoded, databased and frozen using a two-stage process; 24 h at − 80 °C and then in liquid nitrogen (vapour phase at − 180 °C) tanks at the central laboratory which, when full, were transported to a Human Tissue Authority licensed, International Organization for Standardization accredited commercial cryofacility (Fisher Bioservices, UK). The subset of samples selected for the current study were thawed and immediately aliquoted into 2D barcoded tubes for ELF testing.

The ELF test was performed at the Central ELF laboratory (iQur, London). Serum samples were analysed for levels of HA, TIMP-1 and PIIINP using the proprietary assays developed for the ELF test by Siemens Healthineers Inc. These assays are magnetic particle separation immunoassays, and samples were analysed on an ADVIA Centaur® immunoassay system (Siemens Healthineers Inc., Tarrytown, NY, USA). Results were entered into the manufacturer’s published algorithm to derive an ELF score.

### Statistical analysis

We have previously evaluated potential confounding risk factors in univariate Cox proportional hazards models to determine their individual associations with liver disease, confirming that deprivation score and self-reported hypertension, heart disease, hypercholesterolaemia and diabetes were all independently associated with risk [10].

#### Analysis of recruitment samples

Cox proportional hazards models were generated to evaluate the association of ELF score at recruitment with LRE. Univariate models were produced, and then adjusted for covariates listed above for ELF score thresholds of 9.8, as recommended by the manufacturer in the ELF instructions for use (Siemens Healthineers) and 10.51, a threshold recommended in the National Institute for Health and Care Excellence (NICE) Guidance No.49 on management of NAFLD and used in the stratification of patients with alcohol related liver disease [21]. This threshold was selected by NICE based on the performance of the ELF test in predicting liver fibrosis stage using liver biopsy as the reference in a paediatric cohort of NAFLD [5].

#### Analysis of serial samples

To evaluate ELF as a time-dependent variable (and to minimise immortal time bias), time-dependent Cox analysis was performed using the same ELF thresholds [22]. Immortal time bias refers to the period of follow-up during which the study outcome could not have occurred. It occurs with the passing of time before a participant is subject to the exposure or defined level of the covariate (e.g. ELF ≥9.8). The period is considered immortal because participants necessarily had to remain event-free until the time of ‘exposure’ (in this case a high ELF score) to be classified as ‘exposed’. An incorrect consideration of this ‘unexposed’ time period will lead to immortal time bias [23, 24]. In this study, the time during follow-up at which ELF reached the threshold was assumed to be the time of the first sample in which ELF was measured at or above that threshold. Both univariate and adjusted models were produced.

### Exploration of ELF thresholds

Performance of the ELF test was further evaluated by deriving the area under the receiver operator characteristic (AUROC) curves (with 95% confidence intervals (CI)). Optimal cut-off values for discriminating between cases and controls were determined by identifying the ELF score at the point of maximum sensitivity and specificity on the ROC curve, by calculating the Youden Index. Diagnostic odds ratios of these single threshold models were calculated.

The potential clinical utility of the ELF test in this population was further evaluated by selecting an upper ELF threshold with high specificity, therefore high positive predictive value to ‘rule in’ an event and a low threshold with high sensitivity and therefore high negative predictive value to ‘rule out’ an event, at a range of sensitivities and specificities. The proportion of subjects in which a second test would be needed to identify LRE was calculated for each model.

## Results

**Fig. 1:**
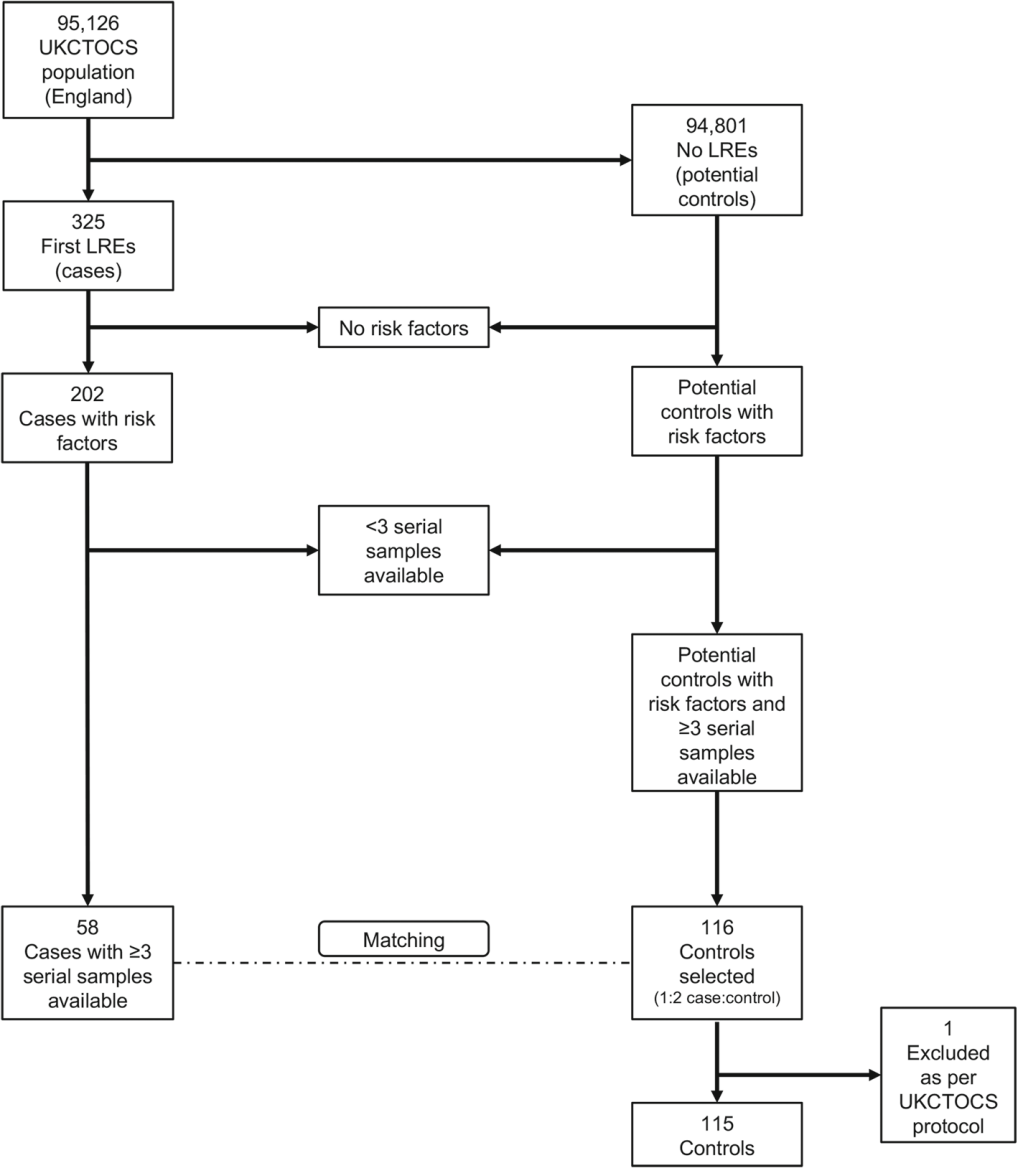
Derivation of the study cohort. Cases were included if a recruitment sample and two subsequent samples were available. For each case, the recruitment and two subsequent samples were selected (with the third sample taken at a time point at least 6 months before the LRE). Samples from each control were selected at the closest equivalent time points to the respective cases

Of the 95,126 participants from UKCTOCS studied (see Trembling et al., 2018 for details of this cohort) [10], 325 participants experienced a first LRE, of which 58 had recorded risk factors and at least three serial serum samples stored. These cases were matched to 116 controls as described above. Of the 522 samples selected, one control (3 samples) was excluded as per UKCTOCS protocol due to a diagnosis of ovarian cancer during follow-up, and one subsequent sample from a case was not available. Derivation of the study population is shown in Fig. 1.

### Baseline characteristics

Baseline characteristics of the study cohort are presented in Table 1. Median recruitment age was 61 years (range 52–74). High alcohol use was reported by 19% and BMI ≥ 25 kg/m^2^ in 88%. Median time to LRE / censor was 8.5 years (range 0.5–11.4).

**Table 1:** Baseline characteristics of study participants and comparisons between cases and controls. Data are presented for the study cohort, categorised in to cases and controls, for mean deprivation score, numbers of self-reported comorbidities, and WHO BMI categories. Tests of statistical differences were applied

Characteristic		Cases	Controls	All participants	***p*** value^**†**^
Participants, *n*		58	115^‡^	173	
Age at recruitment, median (range)		60.9 (51.6–74.3)	61.5 (51.8–74.2)	61.0 (51.8–74.3)	0.8501^a,d^
IMD, mean (SD)		25.55 (17.03)	19.86 (15.61)	21.8 (16.3)	0.031^a^
Hypertension, *n* (%)		26 (44.8)	39 (33.9)	65 (37.6)	0.162^b^
Heart disease, *n* (%)		8 (13.8)	13 (11.3)	21 (12.1)	0.636^b^
Hypercholesterolaemia, *n* (%)		22 (37.9)	32 (27.8)	54 (31.2)	0.176^b^
Type 2 diabetes, *n* (%)		11 (19.0)	5 (4.3)	16 (3.4)	0.002^b^
Smoker, *n* (%)^e^		33 (57)	38 (33) 13 missing	71 (44.4) 13 missing	0.016^b^
Stroke, *n* (%)		1 (1.7)	0 (0.0)	1 (0.6)	0.335^c^
BMI (kg/m^2^) *n*, (%)	< 25	7 (12.1)	15 (13.0)	22 (12.7)	0.856^a,d^
	25 - < 30	33 (56.9)	65 (56.5)	98 (56.6)	0.963^a,d^
	≥30	18 (31.0)	35 (30.4)	53 (30.6)	0.936^a,d^
Alcohol (units/week) *n,* (%)	None	18 (31.0)	35 (30.4)	53 (30.6)	0.936^a,d^
	< 1–10	29 (50.0)	58 (50.4)	87 (50.3)	0.957^a,d^
	11–15	7 (12.1)	14 (12.2)	21 (12.1)	0.984^a,d^
	16–20	2 (3.4)	4 (3.5)	6 (3.5)	0.992^a,d^
	≥21	2 (3.4)	4 (3.5)	6 (3.5)	0.992^a,d^

*WHO* World Health Organization, *BMI* body mass index, *IMD* Index of Multiple Deprivation

^†^ at the 5% level. ^‡^ One control excluded as per UKCTOCS protocol

^a^ Independent sample t-test; ^b^Pearson’s Chi-square test; ^c^ Fisher’s exact test; ^d^ Matched variable; ^e^ variable excluded from regression analyses

As per the matching strategy, there was no significant difference in age and there were no significant differences in the proportions of each BMI group and of each alcohol group between the cases and control groups.

The most prevalent comorbidity was hypertension (37%), followed by hypercholesterolaemia (31%), heart disease (12%), type 2 diabetes (3%) and stroke (0.6%). There were significantly more self-reported diagnoses of type 2 diabetes in the cases compared to controls, but there was no significant difference in prevalence of hypertension, heart disease or stroke between groups. There was a significantly higher mean deprivation score in the cases.

### Outcomes

Median interval from recruitment sample to a first presentation of a LRE in cases was 3.8 years (interquartile range 1.5). In controls, median follow-up with no event was 9.8 years (interquartile range 2.1).

The most common ICD-10 code for the study definition of LRE was K76, ‘other diseases of the liver’. When cases with LREs coding for complications of liver disease (I85, Z944 and / or C22.0) were compared to cases with any other LRE code, mean recruitment ELF score, first subsequent ELF score and second subsequent ELF score were not significantly different (9.409 v 9.350, *p* = 0.890; 9.540 v 9.966, *p* = 0.341; 9.646 v 10.161, *p* = 0.204, respectively). The LREs for the cases are shown in Additional file: Table S1.

### ELF scores

**Fig. 2:**
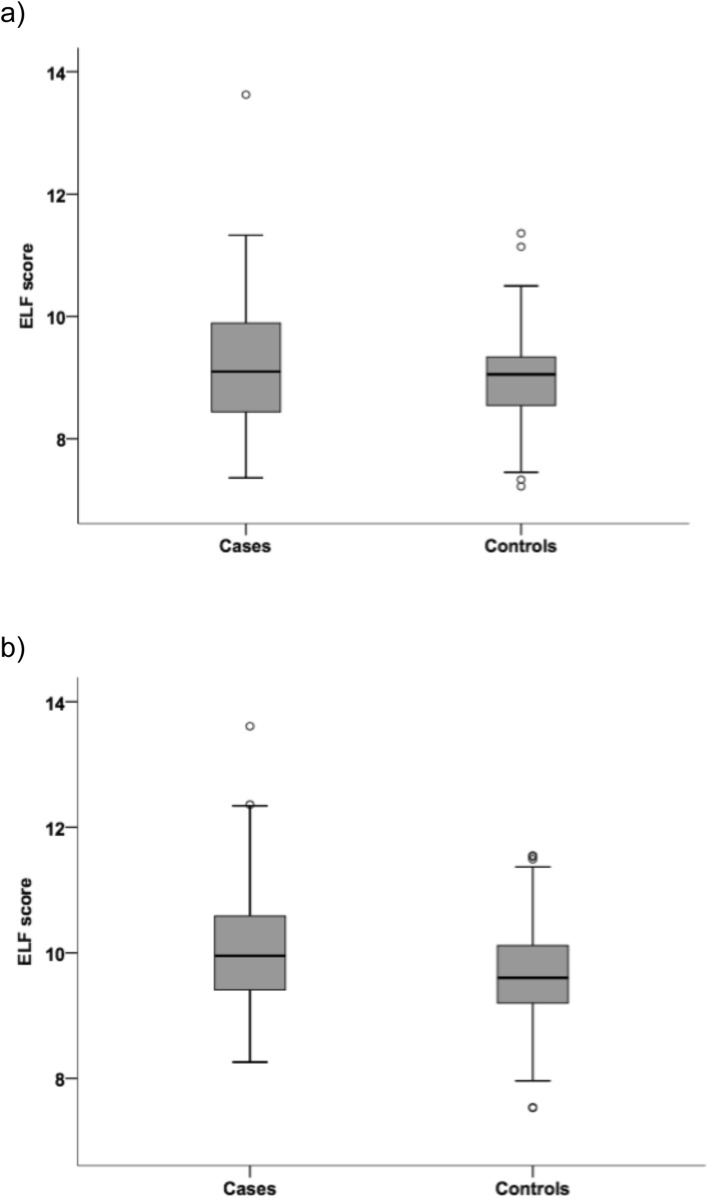
Box plots for ELF scores in **a** cases and **b** controls. Plots show median ELF scores, 25th and 75th percentiles, and minimum and maximum values (outliers are also shown)

Mean ELF scores for recruitment, subsequent samples and combined subsequent samples are shown in Table 2 and median values for recruitment and the second subsequent samples in Fig. 2. The mean concentrations for the three components of the ELF assay for cases and controls are shown in Additional file: Table S2. The mean ELF score in the recruitment samples was higher in the cases compared to the controls (9.36 and 8.96, respectively).

**Table 2:** Mean ELF scores for cases and controls in recruitment samples, subsequent samples and in the combined subsequent samples. Numbers of participants in each group are shown with corresponding mean ELF test score, for first sample (recruitment samples), subsequent sample 1 (second samples) and subsequent sample 2 (third samples)

Sample type	Case / control		***p*** value^**†**^
	Cases^a^	Controls^b^	
Recruitment sample			
Number of participants	58	115	0.007
Mean ELF score (SD)	9.355 (1.136)	8.959 (0.743)	
Subsequent sample 1			
Number of participants	58	115	0.030
Mean ELF score (SD)	9.901 (1.198)	9.588 (0.798)	
Subsequent sample 2			
Number of participants	57	115	0.002
Mean ELF score (SD)	10.143 (1.017)	9.669 (0.807)	
Combined subsequent samples			
Number of participants	115	230	< 0.001
Mean ELF score (SD)	10.022 (1.138)	9.628 (0.802)	

*ELF* enhanced liver fibrosis, *SD* standard deviation

^†^ at the 5% level

^a^ One subsequent sample not available for testing

^b^ One control excluded from analysis as per UKCTOCS protocol due to a diagnosis of ovarian cancer

Fifteen (25.9%) cases had a recruitment ELF score of ≥9.8 compared with 14 (12.2%) controls. Nine (15.5%) cases had a recruitment ELF score of ≥10.51 compared to 2 (1.7%) controls.

When the time to first LRE in the cases was divided at the median, the mean recruitment ELF score was higher in the group with a shorter time to event compared to the group with the longer time to event (9.45 (standard deviation 1.34) and 9.26 (standard deviation 0.91), respectively (*p* = 0.15)).

**Fig. 3:**
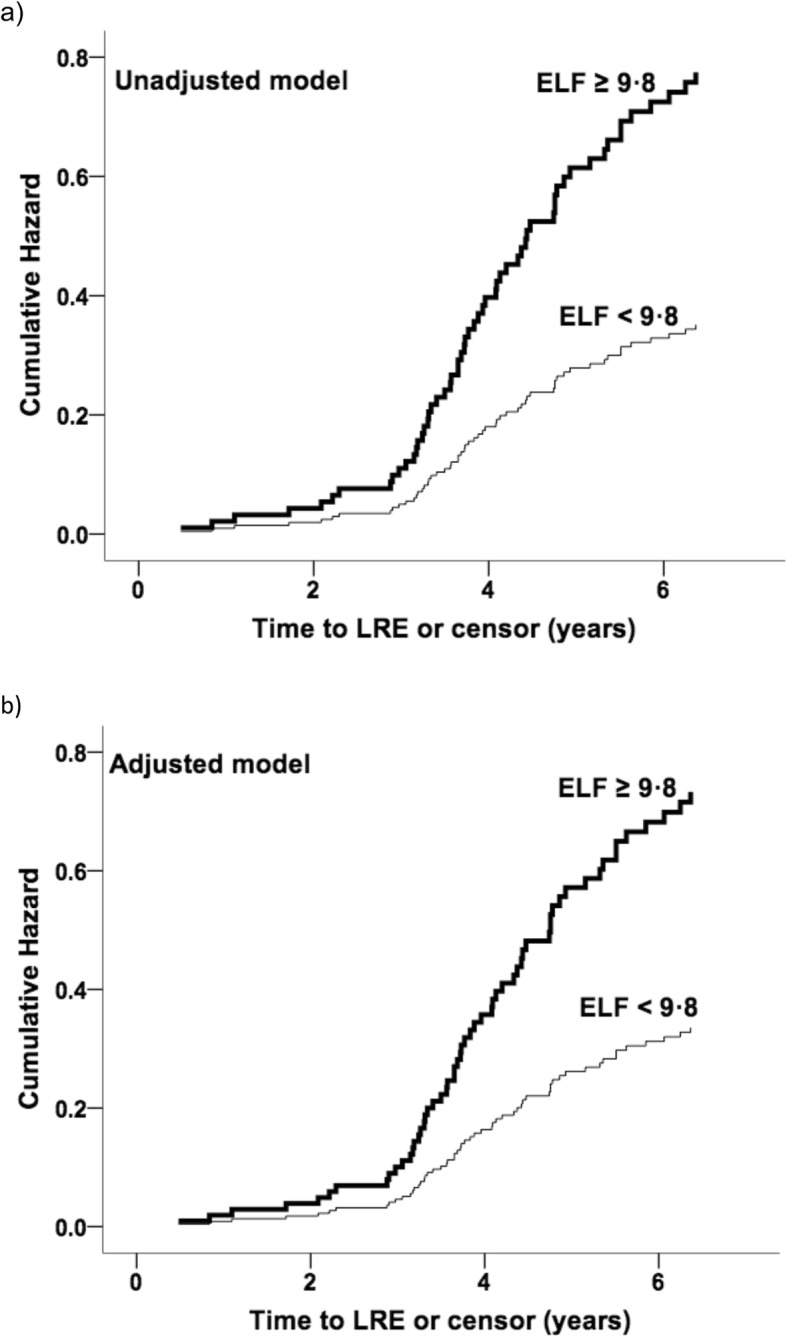
Cumulative hazards for LRE using ELF threshold of 9.8. Cumulative hazards plots for liver-related event for ELF threshold of 9.8 are shown, for **a** an unadjusted model and **b** a model adjusted for deprivation, hypertension, heart disease, hypercholesterolaemia and diabetes

**Fig. 4:**
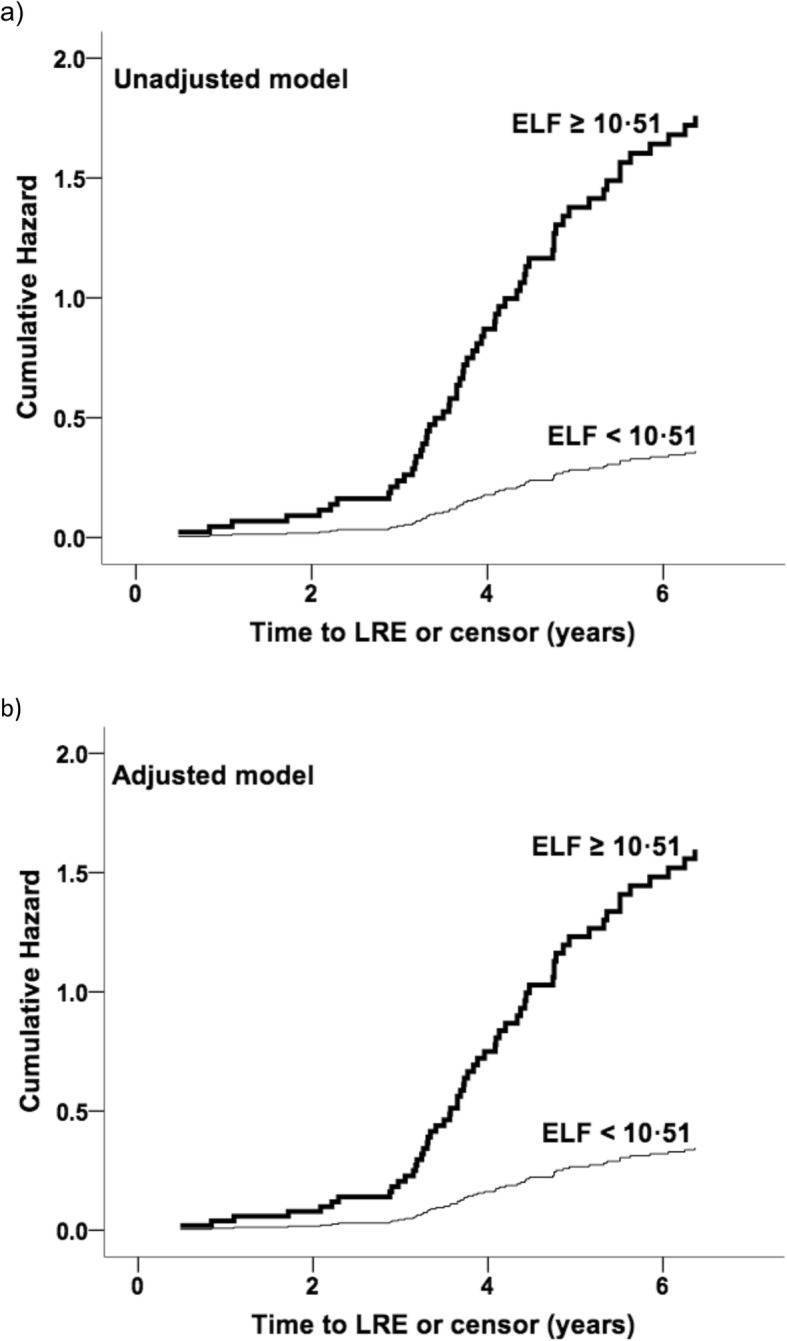
Cumulative hazards for LRE using ELF threshold of 10.51. Cumulative hazards plots for liver-related event for ELF test threshold of 10.51 are shown, for **a** an unadjusted model and **b** a model adjusted for deprivation, hypertension, heart disease, hypercholesterolaemia and diabetes

HR estimates for ELF at recruitment are shown in Table 3. With an ELF threshold of 9.8 to identify advanced fibrosis or cirrhosis (Metavir histological stage F3/F4) [25], HR for LRE was 2.21 in the unadjusted model and 2.18 in the adjusted model. At the threshold of 10.51, HR in the unadjusted model was 4.88 and in the adjusted model HR was 4.62. Cumulative hazard estimates for both models are shown in Figs. 3 and 4.

**Table 3:** Hazard ratio estimates for liver-related event at ELF thresholds of 9.8 and 10.51. Hazard ratio estimates are presented using standard Cox proportional hazards and using time-dependent Cox analysis for liver-related event, at two ELF thresholds. Hazard ratio estimates are shown in unadjusted models and in models adjusted for deprivation, hypertension, heart disease, hypercholesterolaemia and diabetes

ELF threshold	Unadjusted / adjusted	Cox		Time-dependent Cox	
		HR (95% CI)	***p*** value^†^	HR (95% CI)	***p*** value^†^
9.8	Unadjusted	2.205 (1.224–3.971)	0.008	1.854 (1.092–3.148)	0.022
	Adjusted	2.184 (1.189–4.013)	0.012	1.804 (1.041–3.126)	0.035
10.51	Unadjusted	4.880 (2.374–10.029)	< 0.0001	1.935 (1.104–3.391)	0.021
	Adjusted	4.617 (2.115–10.081)	< 0.0001	2.053 (1.157–3.644)	0.014

*ELF* enhanced liver fibrosis, *HR* hazard ratio, *CI* confidence interval

^†^ at the 5% level

In the time-dependent Cox models, at an ELF threshold of 9.8 HRs are 1.85 and 1.80 in the unadjusted and adjusted models, respectively, and at a threshold of 10.51, HRs are 1.94 and 2.05 in the unadjusted and adjusted models, respectively.

### Exploration of ELF thresholds and clinical utility models

The AUROC for recruitment ELF predicting event was 0.583 (95% CI 0.487–0.678), and for second subsequent ELF 0.613 (95% CI 0.521–0.705).

The Youden Index for recruitment ELF was 0.214, with an optimal calculated ELF threshold of 9.535 (sensitivity 37.9%, specificity 83.5%). The Youden Index for second subsequent ELF was 0.228, with an optimal calculated ELF threshold of 10.325 (sensitivity 39.3%, specificity 83.5%). In these ‘fully assigned’ models, the diagnostic accuracy using recruitment ELF would be 68.2%, and using the second subsequent ELF score 69%. The diagnostic odds ratios for each model are 3.1 and 3.3, respectively.

The calculated dual thresholds at a range of sensitivities and specificities are shown in Table 4, along with diagnostic accuracy, misclassification and indeterminate rates. In these models, compared to single threshold models, the misclassification rates are lower, but at the expense of diagnostic accuracy and need for a second test to classify indeterminate subjects.

**Table 4:** Diagnostic performance indices of ELF for identifying LRE using thresholds with sensitivity and specificity of 80, 85 and 90%. Data are presented showing ELF thresholds with sensitivity and specificity values of 80, 85 and 90%, with data-derived diagnostic accuracy (proportion of true positives and true negatives), misclassification rate (proportion of false negatives and false positives) and the rate of indeterminate cases (ELF score between the thresholds)

Sensitivity and specificity values	ELF thresholds	Sensitivity (%)	Specificity (%)	Diagnostic accuracy (%)	Misclassification rate (%)	Indeterminate (%)
Recruitment samples						
80%	8.350	81.0	19.1	26.0	19.7	54.3
	9.425	39.7	80.0			
85%	8.270	84.5	16.5	26.0	19.7	54.3
	9.750	27.6	85.2			
90%	8.160	91.4	14.8	26.0	19.7	54.3
	9.895	24.1	90.4			
Second subsequent samples						
80%	9.365	80.4	34.8	36.4	19.1	44.5
	10.255	41.1	80.9			
85%	8.975	85.7	14.8	36.4	19.1	44.5
	10.540	28.6	86.1			
90%	8.945	89.3	13.9	56.6	28.3	15.1
	10.835	17.9	90.4			

*ELF* enhanced liver fibrosis

## Discussion

This prospective case-control study demonstrates the association of the ELF test score with liver-related outcomes in a general population of postmenopausal women with risk factors for liver disease in the form of high BMI, high alcohol consumption or both. ELF scores were higher in those participants who subsequently experienced a liver-related event earlier. Time to event analysis demonstrated an association between ELF and LRE, with a hazard ratio of 2 compared to women who do not experience LRE. To the best of our knowledge, this is the first study to evaluate the performance of the ELF test to predict a range of LREs in a general population.

The clinical utility models indicate that the ELF test may have a role in diagnosing liver disease in this low prevalence population, using a single threshold. The AUROC in our study was not highly predictive, and further population studies are required.

### Strengths and limitations

Strengths of this population-based study include the prospective design and the independence of data capture for outcomes. This study used ICD-10 codes for CLD that have been used in other studies of cirrhosis, however in an attempt to maximise identification of liver disease we also included codes relating to clinical consequences of advanced cirrhosis. Evaluation of numerous possible confounders including self-reported comorbidities and socioeconomic status minimised bias. Although the number of cases of liver related events was only 58 it is important to recognize that this was the total number of incident cases occurring in a cohort of 95,126 women sampled for this study and so the cases represent a comprehensive sample of participants developing LRE.

ELF tests were performed in one central laboratory, ensuring quality control and consistency, using the proprietary ELF assays. The stability of the ELF test, when applied to samples exposed to a range of common storage conditions, has been demonstrated [26].

Limitations of this study include the reliance of self-reporting of height and weight and co-morbidities. There is evidence, however, of good reliability of self-reporting height and weight from other studies [27–30], for example in a longitudinal study examining agreement between self-reported and measured height, weight and body mass index in older people [31]. Several studies have demonstrated good accuracy in recalled weight, with some data indicating underestimation in those with higher BMI [32–35].

As previously discussed, the UKCTOCS alcohol categories do not align with the CMO’s threshold for hazardous drinking of 14 units / week and therefore including the UKCTOCS threshold of 11–15 units / week may have over-estimated ‘high alcohol use’; however excluding this category in this study would have risked excluding some women with hazardous alcohol consumption.

It is possible that reliance on ICD-10 codes to define events may lead to errors due to mis-coding. We interrogated three independent sources in an attempt to reduce risk of non-coding. Further, HES data may not capture clinical events in a number of areas of healthcare, including the private sector. There is no clear definition for liver disease and consequently, due to large variation in data definitions, comparing incidence between studies is difficult. The problem facing clinicians is that nearly 50% of individuals with liver disease only receive the diagnosis when they present to hospital with a decompensating event [36]. In the community setting the focus must be on identifying liver disease not only at the point of a clinical event but before this where intervention may be more effective. By selecting a group of codes for our data definition, we are contributing to the heterogeneity of definitions in this area. Further work is required to find agreement amongst investigators.

Ninety seven percent of the UKCTOCS population was white. This ethnic homogeneity may have implications for general applicability of the data, in addition to the use of ‘normal’ ELF score ranges. Finally, an evaluation of the ELF test in a secondary care population in Australia reported a positive correlation with age, not seen in the original ELF studies [37]. More work may be required to determine whether age-specific ranges are required in the general population.

### Other studies

The incidence of CLD continues to rise [19, 38–40] and the asymptomatic nature of liver fibrosis progression, leading to cirrhosis results in individuals often presenting with life-threatening features of decompensation in the form of ascites, variceal haemorrhage, hepatic encephalopathy, liver failure or hepatocellular carcinoma [36]. Noninvasive evaluation of liver fibrosis is now established in clinical practice but remains largely confined to secondary and tertiary care settings where it is applied to patients with known or suspected liver disease. There is an urgent need to identify liver disease and the risk of progressive fibrosis in primary care, not least in those with risk factors, where the reliance on measurement of serum liver enzymes may be falsely reassuring [41–43].

Transient elastography (TE) has been the most extensively investigated and independently validated non-invasive test in the general population, with smaller numbers of studies evaluating and validating serum-based markers [44]. However, there is a lack of consensus on thresholds for levels of liver disease, both between tests and within the same test. This results in a wide variation in prevalence estimates. Furthermore, although elastography is a relatively rapid test which produces an instant result, automated blood tests, like the ELF test, can be included with other routine blood tests in primary care and, unlike TE, require no training (which would be more difficult to provide in primary care) and have a lower failure rate compared to TE [45].

TE is operator dependent and although good inter-operator variability in performance has been reported [46, 47], this is reduced at lesser stages of fibrosis and in individuals with hepatic steatosis, high BMI and in particular waist circumference. In its development of guidelines for management of NAFLD, NICE performed an extensive health economic comparison of non-invasive modalities, concluding that the ELF test was the most cost-effective test in this context [48]. More recently a prospective study in primary care has shown that the ELF test improved the detection of liver disease and in conjunction with the Fibrosis-4 (Fib-4) score, reduced inappropriate referrals of patients with NAFLD to secondary care [49]. The choice of modality utilised in a community-based setting is likely to be influenced by local expertise and experience and the prevailing capital and processing costs.

A recent meta-analysis collected data on TE values in healthy individuals identifying 26 studies and a total of 16,082 participants [50]. The mean liver stiffness in non-obese individuals was 4.68 kPa, with increased stiffness measurements in individuals with diabetes, increasing waist circumference, obesity, elevated serum transaminases or hypertension. Dedicated studies within primary care are, however, required.

A community-based study of participants with risk factors for liver disease comprising excess alcohol use, type 2 diabetes or elevated serum transaminases used simple serum marker algorithms with high negative predictive values to rule out significant liver fibrosis. Those with results indicating liver disease were invited to attend for TE. 12.1% had a normal initial test and of those with a valid elastography result, 27% had elevated liver stiffness [43].

A large study based in primary care examined the natural history of standard ‘liver function tests’ (LFT) measured in over 95,000 patients with no liver disease, followed up for a median of 3.7 years [41]. 1.14% developed liver disease and at least one abnormality within the LFT panel was predictive of developing liver disease, for example the HRs for mild ALT and severe ALT rises were 4.23 and 12.67, respectively. Health economic analyses indicated that the most cost-effective strategy in those with abnormal LFTs with no obvious liver disease was to re-test in primary care and in those with high risk neither re-testing nor secondary care referral dominated. In a group of individuals from the general population participating in the National Health and Nutrition Examination Survey (NHANES) III survey, serum markers were applied to those with NAFLD and followed up for a median of 14.5 years. Increasing NAFLD fibrosis score, aspartate aminotransferase (AST) to platelet ratio (APRI) and Fib-4 scores were associated with increasing mortality, although the low number of liver related deaths was too small to analyse [51].

The ELF test has been evaluated in a group of obese patients undergoing bariatric surgery and who had suspected NAFLD with a significantly higher ELF score in those with non-alcoholic steatohepatitis (NASH) and / or fibrosis on biopsy compared to those with normal histology or steatosis [52]. Using histology as the reference standard, performance of the ELF test, FibroTest, elastography, and other simple serum marker panels (including APRI and Fib-4) were compared in a primary care cohort with a history of excess alcohol use. Using a cut off value of 10.5, the ELF test diagnosed advanced fibrosis with high accuracy (AUROC = 0.89), with similar performance to FibroTest and elastography, but without test failures, and was more accurate than the simple marker panels [21].

The ELF test has been shown to predict clinical outcomes in mixed liver disease [9, 37], and in disease-specific populations, for example primary sclerosing cholangitis [53] and chronic hepatitis C [54]. A study in the general population evaluated the performance of the ELF test to predict development of hepatocellular carcinoma, demonstrating that an ELF score of ≥9.89 had an odds ratio of 25 for predicting an event [55].

Several studies have defined the normal ELF score in healthy populations. In a South Korean cohort where heart disease, diabetes, metabolic syndrome, hepatitis B, hepatitis C and liver dysfunction were excluded, the ELF test score in females was between 5.89 and 8.67 [56]. In a study using serum samples from 400 blood donors, the ELF test score in females was found to be between 6.6 and 9.3 [57]. In both studies, the average ELF score was higher in males. Our data adds to the understanding of normal ranges of ELF, showing a higher ELF score in a general population with risk factors, and higher still in those experiencing LRE.

## Conclusion

This study builds on the growing body of evidence supporting the clinical utility of the ELF test in community settings for diagnosis of liver disease. We have demonstrated the association of the ELF test with clinically significant liver-related events in middle-aged women with risk factors. Serum transaminases are inaccurate measures of CLD and the absence of symptoms or signs of early liver disease combined with the increasing rise in liver-related deaths highlight the need for accurate and reproducible tests to detect liver disease in people at risk who may have no symptoms or signs of liver damage. Stratification in those with risk factors is potentially valuable. Our study population is of particular interest. Patterns of alcohol consumption in women are changing, with 16% of women in England consuming above recommended limits and this behaviour is highest in the 55–64 year old group [58]. Further work is required to demonstrate the generalisability of our findings, and to further investigate the predictive ability of ELF, to other community-based populations, but these data indicate that ELF may have a role in the stratification of risk years before the development of clinically apparent liver disease in asymptomatic individuals with common risk factors for chronic liver disease.

## Supplementary information

**Additional file 1: Table S1.** ICD-10 codes and / or death certificate text of first LREs for the cases. **Table S2.** Assay results for individual components of the ELF test and calculated ELF test score for cases and controls.

## Data Availability

The datasets used and/or analysed during this study may be available from the corresponding author on reasonable request.

## Abbreviations

ELF: Enhanced Liver Fibrosis (test)
NASH: Non-alcoholic steatohepatitis
HA: Hyaluronic acid
TIMP-1: Tissue inhibitor of matrix metalloproteinase-1
PIIINP: Aminoterminal propeptide of procollagen type III
CLD: Chronic liver disease
NAFLD: Nonalcoholic fatty liver disease
BMI: Body mass index
LRE: Liver-related event
UKCTOCS: United Kingdom Trial of Ovarian Cancer Screening
HR: Hazard ratio
PRoBE: Prospective-specimen-collection, retrospective-blinded-evaluation
CA125: Cancer antigen 125
NRES: National Research Ethics Service
CMO: Chief Medical Officer
IMD: Index of Multiple Deprivation
ICD: International Classification of Diseases
HES: Hospital Episode Statistics
NICE: National Institute for Health and Care Excellence
(AU) ROC: (Area under the) receiver operator characteristic
LFT: Liver function tests
NHANES: National Health and Nutrition Examination Survey
AST: Aspartate aminotransferase
APRI: AST to platelet ratio
Fib-4: Fibrosis-4
