# Supplementary material for: The Enhanced Liver Fibrosis test is associated with liver-related outcomes in postmenopausal women with risk factors for liver disease
Source: BMC Gastroenterol. 2020 Apr 15;20:104. doi: 10.1186/s12876-020-01251-w (PMC7158048; doi:10.1186/s12876-020-01251-w)
Supplement: Supplementary file 1 — Additional file 1: Table S1. ICD-10 codes and / or death certificate text of first LREs for the cases. Table S2. Assay results for individual components of the ELF test and calculated ELF test score for cases and controls. [file 12876_2020_1251_MOESM1_ESM.docx]

**Additional file**

**The Enhanced Liver Fibrosis test predicts liver-related outcomes in postmenopausal women with risk factors for liver disease**

Paul M Trembling, Sophia Apostolidou, Aleksandra Gentry-Maharaj, Julie Parkes, Andy Ryan, Sudeep Tanwar, Matthew Burnell, Scott Harris, Usha Menon, William M Rosenberg

**Contents**

Table S1. ICD-10 codes and / or death certificate text of first LREs for the cases

Table S2. Assay results for individual components of the ELF test and calculated ELF test score for cases and controls

**Table S1. ICD-10 codes and / or death certificate text of first LREs for the cases**

The number of codes / death certificate text results is higher than the number of LREs (58) as some participants had more than one code when presenting with first LRE. Numbers of participants with codes of interest are divided by source of the code (hospital admission (HES), outpatient appointment (HES), cancer registration (ONS) and death certification)

| **Source** | **Code or text** | **Number of participants (% of those with LRE)** |
| --- | --- | --- |
| Hospital admission | K70 | 6 (10.3) |
|  | K73 | 2 (3.4) |
|  | K74 | 4 (6.9) |
|  | K76 | 28 (48.3) |
|  | I859 | 1 (1.7) |
|  | Z94.4 | 4 (6.9) |
| Outpatient appointment | Z94.4 | 3 (5.2) |
| Cancer registration | C22.0 | 1 (1.7) |
| Death certificate | K70 | 1 (1.7) |
|  | K74 | 3 (5.2) |
|  | K76 | 5 (8.6) |
|  | Mention of alcoholic liver disease | 2 (3.4) |
|  | Mention of non-alcoholic fatty liver disease | 4 (6.9) |

HES, Hospital Episode Statistics; ICD-10, International Classification of Diseases, Version 10; LRE, liver-related event; ONS, Office for National Statistics

**Table S2. Assay results for individual components of the ELF test and calculated ELF test score for cases and controls**

Mean concentrations for each assay component with corresponding standard deviation, median and interquartile range values, and the mean calculated ELF score

| **Participant type** | | **Assay** | | | |
| --- | --- | --- | --- | --- | --- |
|  |  | **HA**  **(ng/ml)** | **P3NP**  **(ng/ml)** | **TIMP-1**  **(ng/ml)** | **ELF score** |
| Cases | Mean | 93.4 | 9.7 | 244.8 | 9.36 |
|  | SD | 197.2 | 4.4 | 80.9 | 1.14 |
|  | Median | 38.8 | 8.3 | 227.0 | 9.10 |
|  | IQR | 54.4 | 3.9 | 64.1 | 1.53 |
| Controls | Mean | 45.82 | 8.0 | 226.1 | 8.96 |
|  | SD | 37.4 | 2.6 | 46.0 | 0.75 |
|  | Median | 34.7 | 7.6 | 224.1 | 9.05 |
|  | IQR | 32.8 | 2.5 | 52.7 | 0.81 |

ELF, enhanced liver fibrosis; HA, hyaluronic acid; IQR, interquartile range; P3NP, aminoterminal propeptide of procollagen type III; SD, standard deviation; TIMP-1, tissue inhibitor of matrix metalloproteinase-1
